# Supplementary material for: ATP hydrolysis-driven structural transitions within the Saccharomyces cerevisiae Rad51 and Dmc1 nucleoprotein filaments
Source: J Biol Chem. 2025 Jul 26;301(9):110528. doi: 10.1016/j.jbc.2025.110528 (PMC12409439; doi:10.1016/j.jbc.2025.110528)
Supplement: Supplementary Material [file mmc1.docx]

**Title: ATP hydrolysis-driven structural transitions within the *S. cerevisiae* Rad51 and Dmc1 nucleoprotein filaments**

Yeonoh Shin^1^, Stefan Y. Kim^1^ & Eric C. Greene^1^

^1^Department of Biochemistry & Molecular Biophysics, Columbia University Irving Medical Center, New York, NY, 10032, USA

To whom correspondence should be addressed: [ecg2108@cumc.columbia.edu](mailto:ecg2108@cumc.columbia.edu)

**Running title**: CryoEM study of the *S. cerevisiae* Rad51 and Dmc1 nucleoprotein filaments

**This file includes:**

Supporting Figure Legends

Supporting Table 1

Supporting Figures 1 – 6

**Supporting Figure Legends**

**Supporting Figure S1. Cryo-EM image processing pipeline of the Rad51-ADP nucleoprotein filament (A)** Representative micrograph used for the Rad51-ADP nucleoprotein filament data processing. **(B)** Representative 2D classes selected for 3D classification. **(C)** 3D map generation and refinements. **(D)** Final 3D map reconstruction for the Rad51-ADP nucleoprotein filament. **(E)** Fourier shell correlation curve of the final electron density map.

**Supporting Figure S2. Cryo-EM image processing pipeline of the Dmc1-ADP nucleoprotein filament (A)** Representative micrograph used for the Dmc1-ADP nucleoprotein filament data processing. **(B)** Representative 2D classes selected for 3D classification. **(C)** 3D map generation and refinements. **(D)** Final 3D map reconstruction for the Dmc1-ADP nucleoprotein filament.  **(E)** Fourier shell correlation curve of the final electron density map. (F) A potential point of contact between the filaments is shown.

**Supporting Figure S3. Cryo-EM density maps for the ATP- and ADP-bound nucleoprotein filaments.** (A) Rad51 in the ATP bound state. (B) Rad51 in the ADP-bound state. (C) Dmc1 in the ATP bound state. (D) Dmc1 in the ADP-bound state. For each panel, a subsection of the nucleoprotein comprised of six Rad51 or Dmc1 monomers is shown, and the different protein monomers are highlighted in alternating colors.

**Supporting Figure S4. Diagrams of Rad51 and Dmc1 secondary structure topology. (A)** Ribbon diagram of *S. cerevisiae* Rad51 in the ATP-bound state (PDB ID: 9D46)[44] showing the number designation for each alpha helix and beta strand. Also highlighted are the Walker A and B motifs, DNA binding loops L1 and L2, and the FxxA polymerization motif (PM). The bound ssDNA and ATP are shown in black. **(B)** Ribbon diagram of *S. cerevisiae* Dmc1 in the ATP-bound state (PDB ID: 9D4N)[44] showing the number designation for each alpha helix and beta strand. **(C)** Sequence alignment of *S. cerevisiae* Rad51 (UniProt ID: P25454) and Dmc1(UniProt ID: P25453) (residues in red are small, hydrophobic, or both [A, V, I, L, M, F, P]; residues in blue have acidic side chains [D, E]; residues in magenta have basic side chains [K, R]; green residues correspond to all others [H, S, T, N, Q, C, G, Y, W]; “*****”indicates identical residues in Rad51 and Dmc1; “**.**” indicates residues have weakly similar properties; “**:**” indicates strongly similar residues). The sequence was aligned using Clustal Omega [62].

**Supporting Figure S5. Differences in second metal ion positioning for samples containing Ca^2+^. (A)** ATP-binding pocket of *S. cerevisiae* Rad51 highlighting the positions of the two metal ions (PDB ID: 9D46)[44]; the sample was prepared with 20 mM Mg^2+^. **(B)** ATP-binding pocket of *S. cerevisiae* Dmc1 highlighting the positions of the two metal ions (PDB ID: 9D4N)[44]; the sample was prepared with 20 mM Mg^2+^ plus 1.5 mM Ca^2+^. **(C)** Overlay of *S. cerevisiae* Rad51 and Dmc1 highlighting the difference in position of the second divalent metal ion. **(D)** ATP-binding pocket of human RAD51 highlighting the positions of the two metal ions (PDB ID: 8BQ2)[45]; the sample was prepared with 5 mM Ca^2+^. **(E)** Overlay of the ATP-binding pocket from *S. cerevisiae* Rad51 [44] and human RAD51 (PDB ID: 8BQ2)[45]. **(F)** Overlay of the ATP-binding pocket from *S. cerevisiae* Dmc1 [44] and human RAD51 (PDB ID: 8BQ2)[45]. In **(A-F)** Me1 and Me2 are used denote the first and second metal ion binding sites.

**Supporting Figure S6. Loss of L1 contacts with ssDNA in the ADP-bound state. (A)** Overlay of two adjacent Rad51 protomers in the ATP- and ADP-bound states, as indicated, highlighting the location of the L1 DNA-binding loop, α helix 12 and α helix 13. **(B)** Overlay of two adjacent Dmc1 protomers in the ATP- and ADP-bound states, as indicated, highlighting the location of the L1 DNA-binding loop, α helix 12 and α helix 13. **(C)** Close-up view of the Rad51 L1 contacts with the bound ssDNA substrate in the ATP-bound state. **(D)** Close-up view showing the loss of Rad51 L1 contacts with the bound ssDNA substrate in the ADP-bound state. **(E)** Overlay of the Rad51 region encompassing the L1 DNA-binding loop, α helix 12 and α helix 13 in the ATP- and ADP-bound states. **(F)** Close-up view of the Dmc1 L1 contacts with the bound ssDNA substrate in the ATP-bound state. **(G)** Close-up view showing the loss of Dmc1 L1 contacts with the bound ssDNA substrate in the ADP-bound state. **(H)** Overlay of the Dmc1 region encompassing the L1 DNA-binding loop, α helix 12 and α helix 13 in the ATP- and ADP-bound states.

**Table S1. CryoEM parameters.**

|  | **Rad51_ADP**  **PDB: 9NJK**  **(EMD-49485)** | **Dmc1_ADP**  **PDB: 9NJR**  **(EMD-49488)** |
| --- | --- | --- |
| **Data collection and processing** |  |  |
| Microscope | Titan Krios | Titan Krios |
| Voltage (keV) | 300 | 300 |
| Detector | K3 | K3 |
| Magnification | 105,000 | 105,000 |
| Voltage (kV) | 300 | 300 |
| Electron exposure (e**^-^**/Å^2^) | 50 | 58 |
| Defocus range (μm) | -0.75 to -1.75 | -0.75 to -1.75 |
| Pixel size (Å) | 0.855 | 0.823 |
|  |  |  |
| Initial particles picked | 1,748,800 | 1,184,484 |
| Final particles used | 330,601 | 960,233 |
| Map resolution (Å)  FSC threshold | 3.37  0.143 | 2.7  0.143 |
| Map resolution range (Å) | 3.3-3.8 | 2.7-3.3 |
|  |  |  |
| **Refinement** |  |  |
| Model resolution (Å)  FSC threshold | 3.4  0.143 | 2.8  0.143 |
| *Model composition*  Non-hydrogen atoms  Protein residues  Ligands | 13,808  1,793  ADP, MG | 13,886  1,779  ADP, MG |
| *R.m.s. deviations*  Bond lengths (Å)  Bond angles (°) | 0.002  0.589 | 0.004  0.628 |
| *Validation*  MolProbity score  Clash score  Rotamer outliers (%) | 1.56  10.92  0 | 1.78  13.67  0.07 |
| *Ramachandran plot*  Favored (%)  Allowed (%)  Outliers (%) | 98.35  1.65  0 | 97.25  2.75  0 |

**Supporting Figure S1**

**
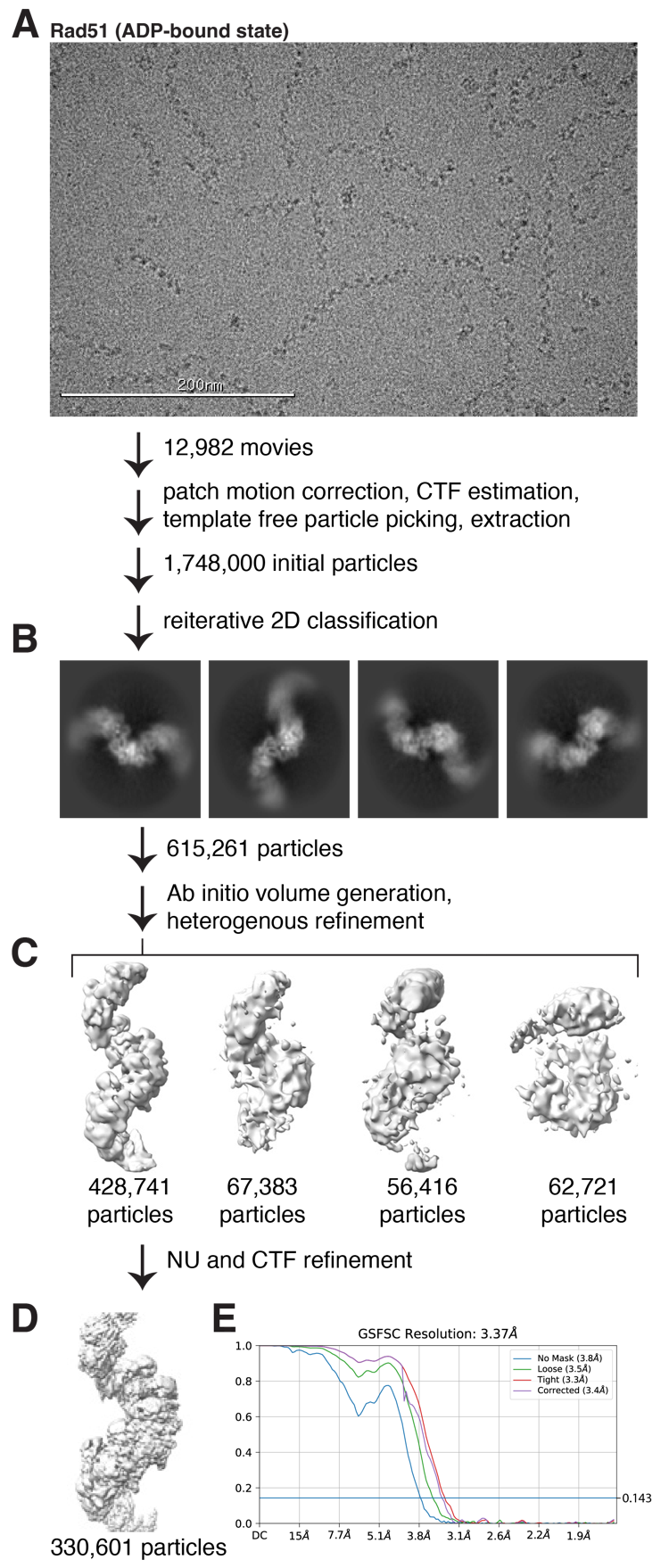
**



**Supporting Figure S2**

**
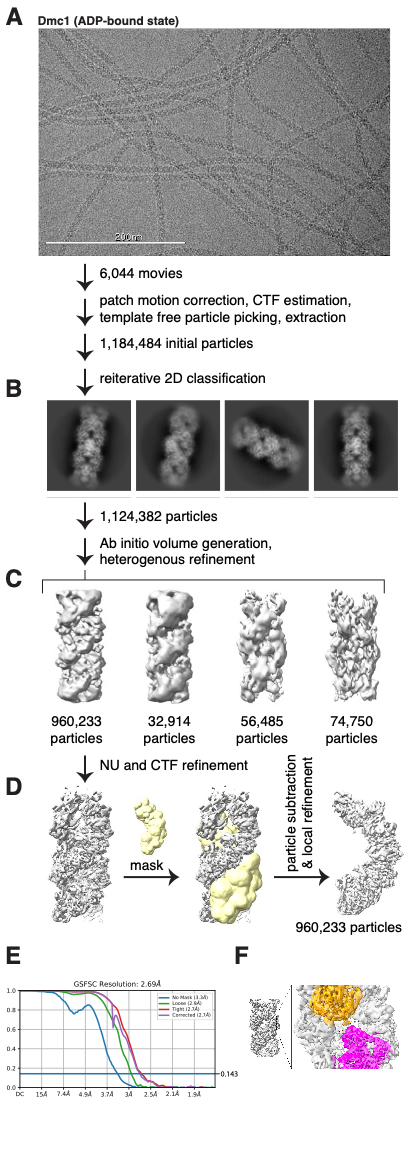
**

**Supporting Figure S3**

**Supporting Figure S4**

**
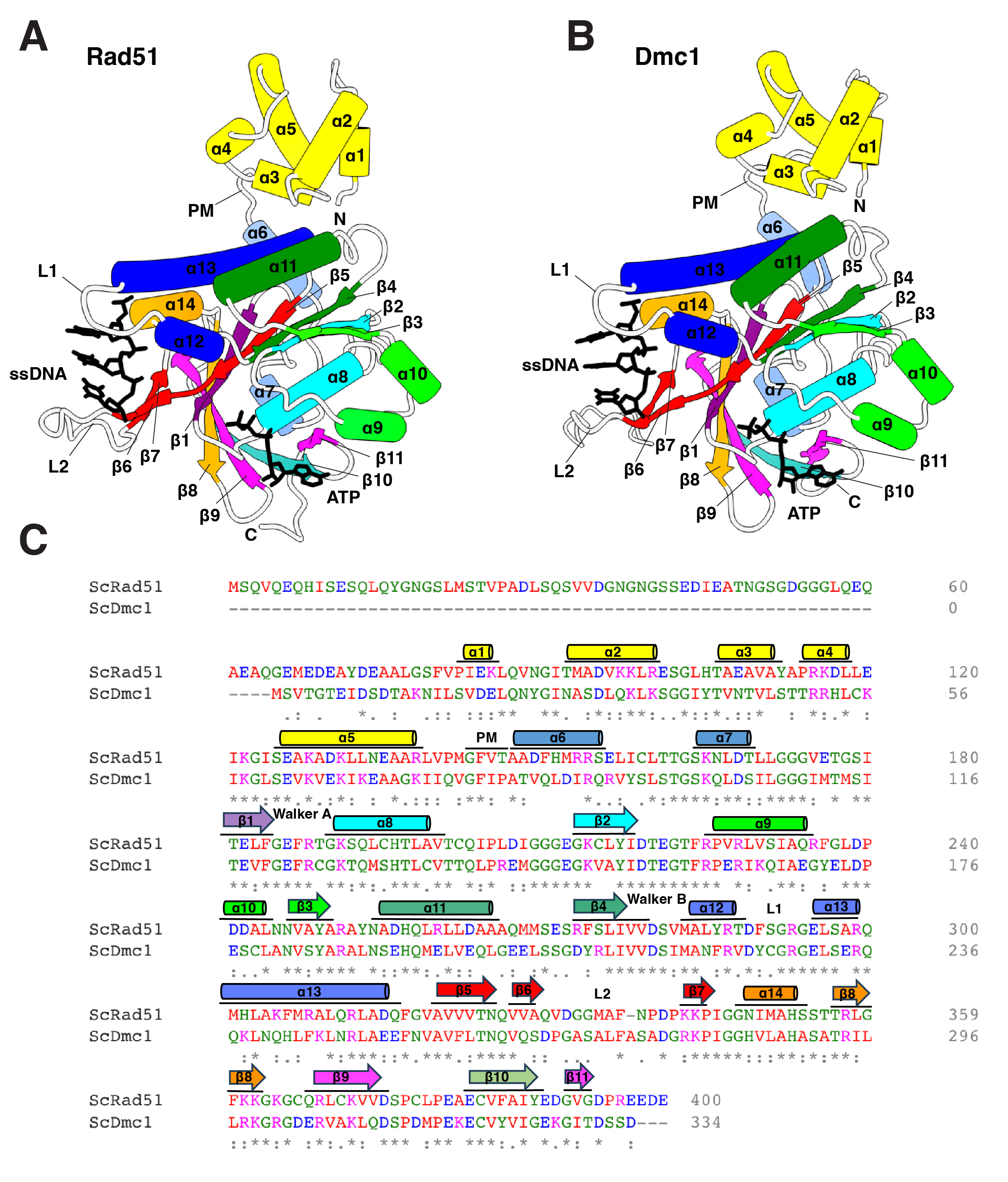
**

**Supporting Figure S5**

**
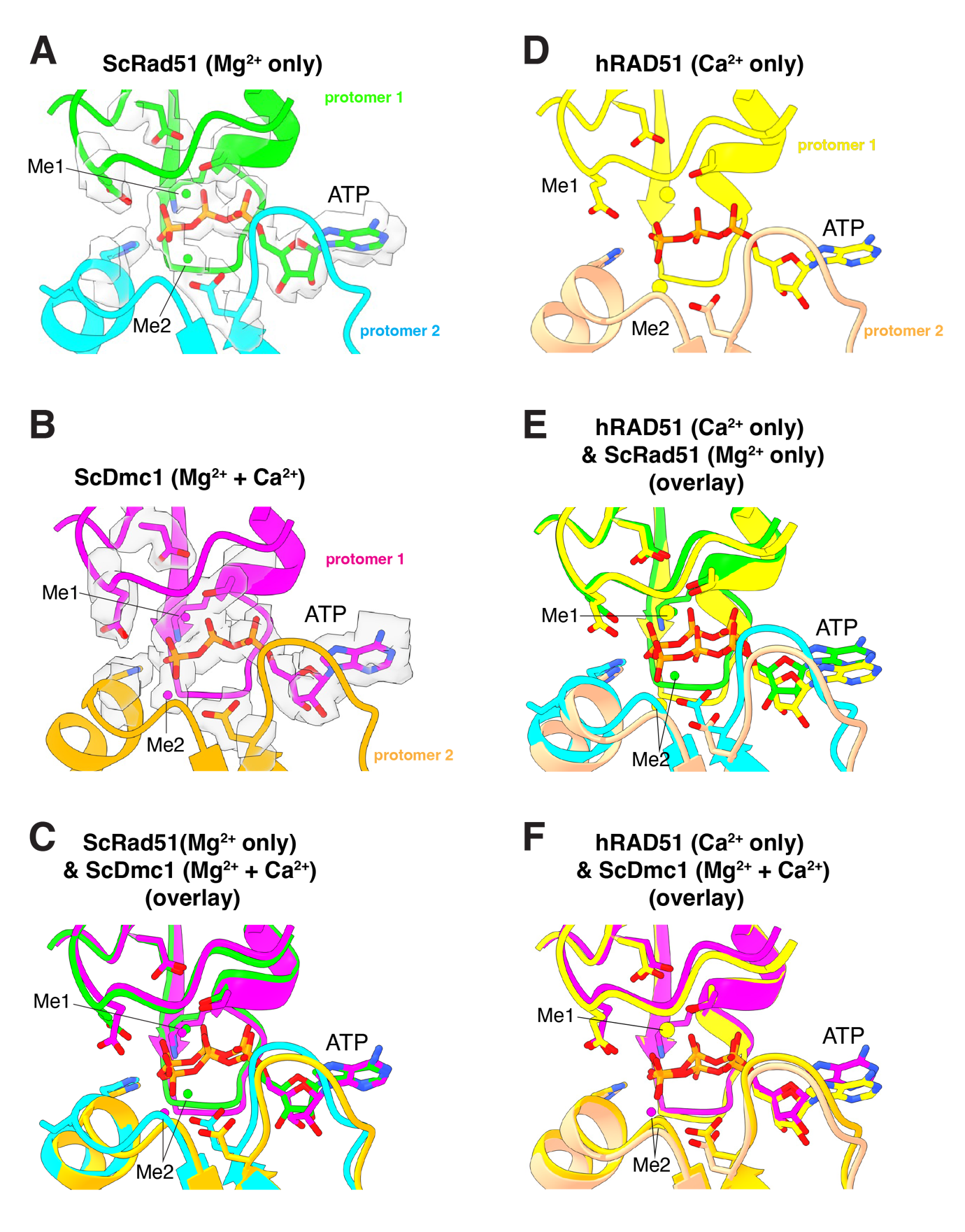
**

**Supporting Figure S6**

**
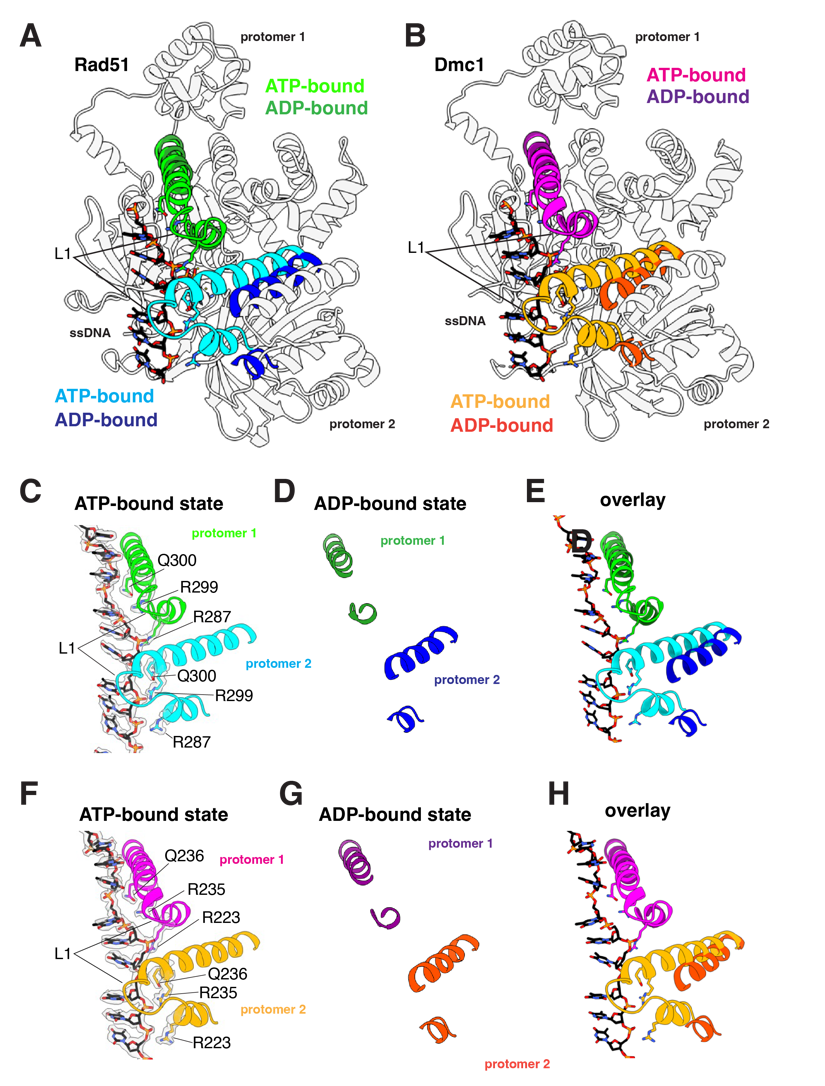
**
